# Determinants of inter-practice variation in ADHD diagnosis and stimulant prescribing: cross-sectional database study of a national surveillance network

**DOI:** 10.1136/bmjebm-2018-111133

**Published:** 2019-02-14

**Authors:** Uy Hoang, Anthony C James, Harshana Liyanage, Simon Jones, Mark Joy, Mitch Blair, Michael Rigby, Simon de Lusignan

**Affiliations:** 1 Department of Clinical and Experimental medicine, University of Surrey, Guildford, UK; 2 Department of Psychiatry, University of Oxford, Warneford Hospital, Oxford, UK; 3 Division of Healthcare Delivery Science/Center for Healthcare Innovation and Delivery Science (CHIDS), Department of Population Health, New York University, Langone Medical Centre, New York, USA; 4 Department of Paediatrics and Child Health, Northwick Park Hospital, Harrow, UK; 5 Section of Paediatrics, Faculty of Medicine, Imperial College London, London, UK; 6 Research and Surveillance Centre, Royal College of General Practitioners, London, UK

**Keywords:** mental health, primary care

## Abstract

Early recognition, identification and treatment of children with attention deficit hyperactivity disorder (ADHD) can reduce detrimental outcomes and redirect their developmental trajectory. We aimed to describe variations in age of ADHD diagnosis and stimulant prescribing among general practitioner practices in a nationwide network and identify child, parental, household and general practice factors that might account for these variations. Cross-sectional study of children aged under 19 years registered within a general practice in the Royal College of General Practitioners (RCGP) Research and Surveillance Centre (RSC) network in 2016, RCGP RSC has a household key allowing parent and child details to be linked. Data from 158 general practices and 353 774 children under 19 were included. The mean age of first ADHD diagnosis was 10.5 years (95% CI 10.1 to 10.9, median 10, IQR 9.0–11.9) and the mean percentage of children with ADHD prescribed stimulant medications among RCGP RSC practices was 41.2% (95% CI 38.7 to 43.6). There was wide inter-practice variation in the prevalence of diagnosis of ADHD, the age of diagnosis and stimulant prescribing. ADHD diagnosis is more likely to be made later in households with a greater number of children and with a larger age difference between adults and children. Stimulant prescribing for children with ADHD was higher in less deprived practices. Older parents and families with more children fail to recognise ADHD and may need more support. Practices in areas of higher socio-economic status are associated with greater prescribing of stimulants for children with ADHD.

## Background and aims

Attention deficit hyperactivity disorder (ADHD) is the most common psychiatric disorder in childhood and adolescents.^1 2^ ADHD is associated with failure in the attainment of many normal developmental milestones and can result in children experiencing school failure, poor family and peer relations, low self-esteem, as well as other emotional, behavioural and learning problems.^3^

Early recognition of this condition is critical as diagnosis and resultant intervention that addresses the wide range of personal, social, educational and occupational needs of children with ADHD can redirect the developmental trajectory of children with this condition.^2^ While current guidance from The National Institute for Health and Care Excellence (NICE) recommends that ‘primary care practitioners should not make the initial diagnosis or start medication in children or young people with suspected ADHD’,^4^ general practitioners (GPs) and other primary care professionals play an important role in identifying children and young people who have persistent behavioural and/or attention problems with at least moderate impairment to secondary care so that opportunities for assessment and referral are not missed.^5^

There is evidence that the threshold for ADHD diagnosis and the prescribing of stimulant medications may be influenced by demographic characteristics of the child,^6^ parental and family characteristics or other broader structural determinants such as socio-economic deprivation.^7 8^ However, the factors which are associated with variations in ADHD diagnosis and prescribing within primary care are largely unexamined.^9 10^

We carried out this study to describe variations in the average age of ADHD diagnosis and prescribing of stimulant medications among general practices who are members of the Royal College of General Practitioners (RCGP) Research and Surveillance Centre (RSC) network^11^; and to identify child, parental, household and general practice factors that might account for these variations.

## Methods

### Subjects and setting

We used information from the RCGP RSC sentinel network database. At the time of this study it hosted a pseudonymised dataset from a nationally representative sample of just under 2 million people registered with a network practices.^11^ It has been used in surveillance of influenza and respiratory disease for over 50 years. Over this period practices have had feedback about their data quality around influenza and respiratory disease—in particular the differentiation of first or new (incident) from follow-up consultations. Feedback about data quality is also provided via a dashboard, that is refreshed weekly^12^ and is good for routine primary care.^13^

UK general practice is suitable for this type of study because it has a registration-based system with patients registered with a single practice. Practices have been computerised since the late 1990s, with pay-for-performance (P4P) introduced in 2004.^14^ Key data are coded,^15^ and this includes diagnoses, therapy, test results and other key data. Additionally, the RCGP RSC has a household key enabling people in the same household to be characterised.^16^

### ADHD diagnosis and stimulant prescribing

We used coded data reported in online appendix 1 as Read codes to extract information on children with a diagnosis of ADHD under 19 years of age between January 1st and December 31st 2016. The age at which the first diagnosis of ADHD was noted for each child, and the average age of ADHD diagnosis within each practice was calculated.

10.1136/bmjebm-2018-111133.supp1 Supplementary data

We used Read codes, and also some CMR brand specific proprietary drug dictionary codes, reported in online appendix 2 to extract information on stimulant prescribing for children with ADHD. A 2016 study found that 94% of prescriptions for children with ADHD was for the stimulant methylphenidate.^17^ The proportion of children with ADHD who were prescribed stimulant medications between January 1st and December 31st 2016 within each practice was calculated.

10.1136/bmjebm-2018-111133.supp2 Supplementary data

### Characteristics of children with ADHD within each practice

We extracted demographic information about children with ADHD including their age, sex, ethnicity and socio-economic deprivation as indicated by their index of multiple deprivation (IMD) score.^18^ The IMD is a measure of relative deprivation for small areas of about 1500 people, termedLower Super Output Areas. It is a combined measure of deprivation based on a total of 37 separate indicators that have been grouped into seven domains, each of which reflects a different aspect of deprivation experienced by individuals living in an area.

An average demographic profile of children with ADHD for each practice was compiled including their mean age, the proportion of female children with ADHD and the proportion of children from the lowest IMD quintile. Racial and ethnic disparities between non-white and white populations has also previously been shown in ADHD diagnosis and treatment,^19^ thus we also present the proportion of children who were non-white with ADHD in practices.

The rate of hyperkinetic conduct disorders (HKCD) for each practice was also calculated separately.

### General practice characteristics

Between August 1st and August 31st 2017 we extracted information about the general characteristics of RCGP RSC practices from summary statistics extracted for each practice, from individual practice websites, the NHS choices website and the General Medical Council (GMC) register.^20^ This includes the practice list size, the percentage of children in the lowest IMD quintile, the total number of children registered with the practice, the total number of GPs in the practice, the gender of GPs in the practice, the average number of years since their medical and specialist general practice qualifications, the average number of qualifications of GPs in the practice and whether the practice was in an urban or rural location.

We collected information about the quality of care provided by each practice from the National General Practice Profiles published by Public Health England, including the total achievement score in the P4P scheme, called the Quality and Outcomes Framework (QOF), as well as the total scores in the clinical and public health management domains of the QOF.^21 22^

### Characteristics of households within each practice

We used a household identifier in each medical record to extract information about the average profile of households in each practice including the proportion of single adult households, the average number of children under 19 years in each household, and the average age difference between adults and children in each household.^16^

### Statistical analysis

We report descriptive statistics generally reporting mean for normally distributed data, but mode, median and mean where there is a skewed distribution of data.

We used a Shapiro-Wilk test^23^ to show that the distribution of age of diagnosis of ADHD between practices was not normally distributed (p value=0.001465). The percentage of children with ADHD prescribed stimulant medications across RCGP RSC practices was normally distributed (p value=0.5807). We then undertook a logarithmic transformation of the mean age of ADHD diagnosis between practices.

Multiple linear regression was then performed to examine the effect of child, general practice and household factors on the percentage of children with ADHD prescribed stimulant medications. The residuals from a multivariate regression of mean age of ADHD on study covariates was positively skewed, so we employed a log transformation of mean age of ADHD.

The regression coefficients from this log-level regression was used to calculate an effect of a unit change in the study co-variates with a percentage change in the average ADHD diagnosis age between practices.

All statistical analysis was undertaken using R release V.3.5,^24^ the Global Validation of Linear Models (V.1.0.0.2) R package by  Edsel Pena was used to test that linear model assumptions were met. 

## Results

### Characteristics of practices within the RCGP RSC network

Data from 158 general practices and 353 774 children under 19 years of age in the RCGP RSC national sentinel practice network were included in the study. 82.9% (131/158) practices were located in an urban location. The mean number of GPs per practice was 7.5 (median=7, mode=2) and the mean number of years since GPs had qualified from medical school was 19.7 years. Each GP within the practice had a mean of more than two specialist qualifications in addition to their medical qualifications

The GP practices within the network achieved a mean 97.7% (95% CI 97.1 to 98.4) total P4P/QOF score overall (median=99.0, mode=98.8425). This compares with a mean total P4P/QOF score of 95.5% (95% CI 95.3 to 95.6) median=97.7, mode=100.0 for all practices in England.

### Characteristics of households within the RCGP RSC network

Information from a total of 803 218 households in the RCGP RSC network showed 23.2% included a child under 19 years of age between January 1st and December 31st 2016. This compares with national census data for 2015 of 29% of households with dependent children under 19 years of age. On average 31.0% (95% CI 29.6 to 32.4) of households with children were occupied by a single adult over 18 years of age. This compares with national census data for 2015 of 25% of single parent households. In households with children, there was an average of just under two children under 19 years of age. This compares with the national census data for 2015 that showed that 40% of families had two dependent children and 15% of families had three or more dependent children Within households in the RCGP RSC network the average age difference between adults and children was 36 years (95% CI 35.7 to 36.3).

### Characteristics of children with ADHD in the RCGP RSC network

**Figure 1:**
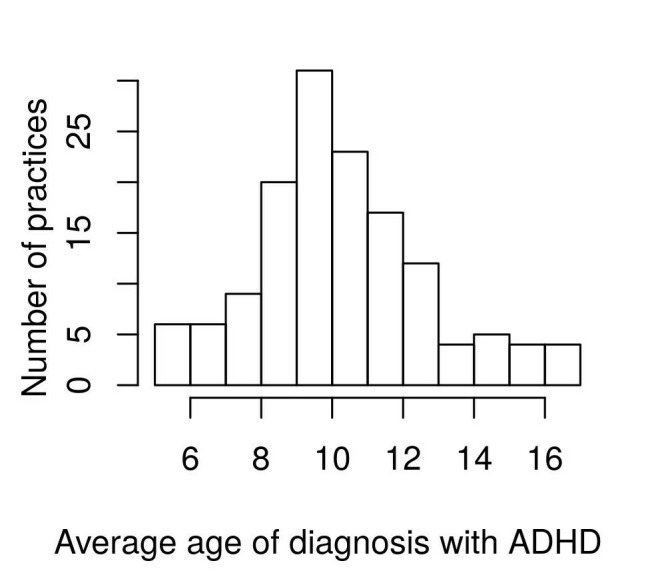
Mean age of first diagnosis of ADHD amongst Royal College of General Practitioners Research and Surveillance Centre practices. ADHD, attention deficit hyperactivity disorder.

3470 children with a coded diagnosis of ADHD within the RCGP RSC network were included in the study. Of these, 649 (18.7%, 95% CI 17.4 to 20.0) were female and 316 (9.1%, 95% CI 8.1 to 10.1) were of non-white ethnicity. There was wide variation in the rate of ADHD diagnosis between practices within the RCGP RSC network from 0.01 to 3.2 per 100 children (inter-quartile range 0.62). See figure 1. The mean rate was 1.0 (95% CI 0.9 to 1.1) and the median was 0.9 per 100 children.

Only 26 children with HKCD were recorded in the RCGP RSC network.

### Age of diagnosis of ADHD

The mean age of first diagnosis with ADHD was 10.5 years (95% CI 10.1 to 10.9, median 10, IQR 9.0–11.9). There was also wide inter-practice variation in the age of first diagnosis of ADHD with the diagnosis being made anywhere between 5 and 17 years of age across RCGP RSC practices.

### Prescribing of stimulant medications in children with ADHD

The mean percentage of children with ADHD prescribed stimulant medications among RCGP RSC practices was 41.2% (95% CI 38.7 to 43.6). There was wide inter-practice variation in prescribing with RCGP practices prescribing stimulant medications for between 5.6% and 77.3% of their patients under 18 years with ADHD.

### Determinants of age of diagnosis and stimulant prescribing in children with ADHD

Multiple regression analysis showed that characteristics of the households in which children with ADHD are raised was a strong predictor of the mean age that children are diagnosed in practices (see table 1).

**Table 1:** Determinants of age of ADHD diagnosis

	Regression estimate (95% CI)	Effect size	Interpretation
Characteristics of households within the practice			
Single parent (>18 years) households	Not statistically significant	Not statistically significant	
Average number of children in the household	0.2759 (0.027774 to 0.524016)	27.59%	For every unit increase in number of children in the household, there is on average 27.6% increase in ADHD diagnosis age within the practice
Average age difference within the household	0.03755 (−0.00109 to 0.0761898)	3.755%	For every unit increase in age difference within practice households, there is 3.8% increase in ADHD diagnosis age within the practice
General practice characteristics			
General practice size	0.00002938 (0.00000730 to 0.00005147)	0.002938%	For every increase in the practice size by 100, there is on average 0.3% increase in ADHD diagnosis age in the practice
Total number of children	−0.0001045 (−0.0001954 to −0.0000136)	−0.001045%	For every unit increase in the number of children in the practice by 100 there is on average 0.1% decrease in ADHD diagnosis age within the practice
Proportion of eligible children—in lowest IMD quintile	Not statistically significant	Not statistically significant	
Mean years since medical qualification of GPs	Not statistically significant	Not statistically significant	
Mean number of specialist qualifications of GPs	Not statistically significant	Not statistically significant	
Urban/rural practice	Not statistically significant	Not statistically significant	
QOF achieved in 2015–2016— overall (%)	Not statistically significant	Not statistically significant	
NHS choices star rating	Not statistically significant	Not statistically significant	
Characteristics of children with ADHD within the practice			
ADHD prevalence rate	Not statistically significant	Not statistically significant	
Hyperkinetic conduct disorder prevalence rate	Not statistically significant	Not statistically significant	
ADHD of non-white ethnicity within the practice (%)	Not statistically significant	Not statistically significant	
Female children with ADHD (%)	Not statistically significant	Not statistically significant	

ADHD, attention deficit hyperactivity disorder;  GP, general practitioner; IMD, index of multiple deprivation; QOF, Quality and Outcomes Framework.

For every unit increase in the average number of children in the household, there was a 27.6% increase of in ADHD diagnosis age within the practice. Equally, for every unit increase in age difference within practice households, there was a 3.8% increase in ADHD diagnosis age within the practice.

Larger practices with less registered children were also more likely to make ADHD diagnosis at an older age, although the magnitude of these effects was much less than the characteristics of the households.

Other characteristics of the general practice including the urban/rural location, the proportion of children in the lowest socio-economic quintile, the qualification of GPs or indicators of the practice clinical care did not significantly affect the inter-practice variations in the age of ADHD diagnosis.

The number of children with ADHD in a practice or the number of children with HKCD did not significantly affect the inter-practice variations in the age of ADHD diagnosis.

Table 2 shows that stimulant prescribing for children with ADHD was significantly determined by the proportion of children in the practice in the lowest IMD quintile, with every unit increase in proportion of children in the lowest IMD quintile in the practice, resulting in 0.1575 less prescriptions for stimulant medication for children with ADHD.

**Table 2:** Determinants of stimulant prescribing in children with ADHD

	Regression estimate (95% CI)	Interpretation
Characteristics of households within the practice		
Single parent (>18 years) households	Not statistically significant	Not statistically significant
Average number of children in the household	Not statistically significant	Not statistically significant
Average age difference within the household	Not statistically significant	Not statistically significant
General practice characteristics		
General practice size	Not statistically significant	Not statistically significant
Total number of children	Not statistically significant	Not statistically significant
Proportion of eligible children—in lowest IMD quintile	−0.1575 (−0.29353 to −0.02157)	For every unit increase in proportion of children in the lowest IMD quintile in the practice, there is 0.1575 less stimulant medication prescribed for children with ADHD
Mean years since medical qualification of GPs	Not statistically significant	Not statistically significant
Mean number of specialist qualifications of GPs	Not statistically significant	Not statistically significant
Urban/rural practice	Not statistically significant	Not statistically significant
QOF achieved in 2015–2016—overall	Not statistically significant	Not statistically significant
NHS choices star rating	Not statistically significant	Not statistically significant
Characteristics of children with ADHD within the practice		
ADHD prevalence rate	Not statistically significant	Not statistically significant
Hyperkinetic conduct disorder prevalence rate	Not statistically significant	Not statistically significant
ADHD of non-white ethnicity within the practice (%)	Not statistically significant	Not statistically significant
Female children with ADHD (%)	Not statistically significant	Not statistically significant

ADHD, attention deficit hyperactivity disorder;  GP, general practitioner; IMD, index of multiple deprivation; QOF, Quality and Outcomes Framework.

## Discussion

### Summary of principal findings

Using data from a nationally representative network of general practices, we found wide inter-practice variation in the prevalence of recorded diagnosis of ADHD.

While most children were diagnosed with ADHD by the age of 10, we also found widespread variations with practices making a diagnosis of ADHD anywhere between 5 and 17 years of age. ADHD diagnosis was more likely to be made later in households with a larger number of children and wider age gaps between adults and children in the households, suggesting that the diagnosis was more likely to be made in families with older parents. Larger practices with less registered children were also more likely to make diagnosis of ADHD at a later age.

Stimulant prescribing for children with ADHD also showed widespread inter-practice variation, with more affluent practices more likely to prescribe stimulant medications for their children with ADHD.

### Strengths and weaknesses of the study

There are a number of strengths of our study including its large sample size of 158 practices from a nationally representative sentinel network, which allows us to examine the impact of a key primary care determinants on the age of ADHD diagnosis, a relatively under-researched area.^9^ In addition, our sample included almost 3500 children with ADHD in primary care which is one of the largest studies to examine factors that affect ADHD diagnosis. This allows us to separately take into take into account the characteristics of the children with ADHD such as sex and ethnicity.

However, our study has a number of limitations including the observational and cross-sectional nature of the study design which does not allow us to draw causal inferences from our results.^25^ In addition as our study focused on practices as the unit of analysis, our results cannot be used to make conclusions about the care of individual patients with ADHD.

There are a number of limitations to our study related to the use of routinely collected data, including the quality of data in primary care records and data about practice characteristics in publicly available sources.

Data from the RCGP RSC sentinel network has been previously appraised and published.^11^ We would expect that our study is limited by the accuracy of the age of ADHD diagnosis which is commonly under-diagnosed, misdiagnosed and undertreated.^26^ It may also be poorly or inaccurately recorded in the patient’s medical record, especially if they change practices. However, we have shown that the prevalence of ADHD among practices in the RCGP RSC network is equivalent to other studies in equivalent populations in the UK.^27^

Assessment of exposure to stimulant medications was not undertaken in this study, thus while we found inter-practice variations in stimulant prescribing practices, we cannot make conclusions about variation in stimulant exposure between practices. This is important as it has been suggested that exposure to stimulant medication, both early initiation of therapy and chronic exposure are important adverse childhood outcomes.^28 29^

We know that the RCGP RSC household key under-estimates household size.^16^ If some people in the same household are registered with a non-RCGP RSC practice they will not be included. Hence RCGP RSC has a greater proportion of single person households compared with census data. When households move, they generally register at the same practice. These factors may affect our interpretation of the impact of households in our study. Additionally, we have no separate information on the impact of families which has been shown to be important in the consequences of ADHD.^26^

The enumeration of practice structural variables for this study relied on the extraction of publicly available data about practices from their own websites, NHS choices and the medical register held by the GMC. The quality of this information, especially its completeness, consistency, timeliness and accuracy has not been validated in published data, and given the number of practices involved in this study we did not attempt to validate the accuracy with individual practice staff.

The outcomes of age of ADHD diagnosis and stimulant prescribing may also be affected by differences in behaviours of individual GPs in practices, however it was not possible to test any clustering of outcomes by individual GPs as there is insufficient details to consistently document activity by GPs in our dataset.

Lastly, we have not included data from child and adolescent mental health services where the majority of diagnoses of ADHD are made. Thus, there is a risk that the diagnosis from secondary care may be missed or there may be a delay in the recording of diagnosis in the primary care record.

### Strengths and weaknesses in relation to other studies, discussing important differences in results

The ADHD prevalence from this study of 1.0%, (0.98 per 100 children, 95% CI 0.95 to 1.01) is similar to previous studies in the UK,^27 30^ although it is lower than figures of global community prevalence published in a recent systematic review of 2%–7%.^31^

Previous studies have found variations in the prevalence of ADHD in community samples and variations in the age of ADHD diagnosis.^31 32^ Using data from a network of general practices we have shown that there also are significant inter-practice variations in ADHD diagnosis, prevalence and stimulant medication prescribing. We have shown that household factors are significant predictors of ADHD diagnosis. This suggests that the wider impact of ADHD symptoms on adults and children within in the same family, rather than the isolated impact on the child, are predictive of recognition of problems in ADHD. This is similar to findings from other studies conducted in the UK.^33^

Sayal and colleagues suggested that parental recognition of problems and views that their child has hyperactivity were associated with greater severity of symptoms. In their sample they found that the majority of parents discuss their concerns with professionals based in education services and few had consulted primary care for these problems.^33^ This highlights a weakness of our study which did not include information from education services.

In contrast stimulant prescribing for children with ADHD was significantly determined by the proportion of children in the practice in the lowest IMD quintile. This is similar to other studies which have found that socio-economic status is an important determinant of stimulant prescribing,^34–38^ although there remains controversy about the direction of this relationship.

### Meaning of the study: possible explanations and implications for clinicians and policy-makers

We have shown that the composition of households is important in the recognition of ADHD. Households with a greater number of children may hinder the parental recognition of an over-active child. The larger age gap between adults and children within households hints at another possible explanation which revolves around the greater experience and situation handling skills of the caregivers, which may result in greater absorption of over-active behaviours and a later ADHD diagnosis in these households.

This would suggest that older parents with more children would benefit from greater support to recognise the symptoms of ADHD and present their children to healthcare services for earlier assessment.

The finding that larger practices with fewer registered children diagnosed children with ADHD at a later age may suggest that GPs in these practices have less confidence in making the diagnosis early and a higher threshold for symptoms to be initially presented in these practices. It could also reflect poorer links with specialist education services or child mental health services for definitive diagnosis in some practices and is an area for further study.

### Unanswered questions and future research

Previous research suggests that parental factors are critical to recognition of ADHD, and our research on households hints at how parental recognition may be influenced by the age and experience of the caregiver.

However, further research is required to understand the barriers and facilitators to early parental recognition, especially the role of statutory services including school health services which are often the first point of call for parents with symptomatic children.

**Summary box**

**What is already known about this subject?**

- Early recognition, identification  and treatment of attention deficit hyperactivity disorder (ADHD) can alter the trajectory of this condition.
- However, the factors which influence variations in ADHD diagnosis within primary care in the UK are not described.

**What are the new findings?**

- This study describes the variation in age of ADHD diagnosis and stimulant prescribing for children with ADHD among a nationally representative sample of English general practices.
- Family factors were significantly predictive of age of ADHD diagnosis in the practice. For every unit increase in the average number of children in the household, there was a 27.6% increase in ADHD diagnosis age within the practice. Equally, for every percentage increase in age difference within practice households, there was a 3.8% increase in ADHD diagnosis age within the practice.
- General practice factors were also significantly predictors of an older age of ADHD diagnosis in general practices, although the magnitude of these effects was much less than the characteristics of the households.
- General practice factors such as a decrease in the proportion of children from the lowest index of multiple deprivation (IMD) quintile in the practice were also significantly associated with increased stimulant prescribing for children with ADHD. For every unit increase in proportion of children in the lowest IMD quintile in the practice, there was 0.1575 less prescriptions for stimulant medication for children with ADHD.
- Patient factors were not significantly predictive of age of ADHD diagnosis or stimulant prescribing for children with ADHD.

**How might it impact on clinical practice in the foreseeable future?**

- Health system factors resulting in wide inter-practice variation in the age of ADHD diagnosis and stimulant prescribing could be identified and addressed to facilitate earlier diagnosis of these children.
- Families with older parents and parents with more children could be offered greater support to recognise the symptoms of ADHD and present their children to healthcare services for earlier assessment.
