# Supplementary material for: Determinants of inter-practice variation in ADHD diagnosis and stimulant prescribing: cross-sectional database study of a national surveillance network
Source: BMJ Evid Based Med. 2019 Feb 14;24(4):155–61. doi: 10.1136/bmjebm-2018-111133 (PMC6678046; doi:10.1136/bmjebm-2018-111133)
Supplement: Supplementary data [file bmjebm-2018-111133supp001.pdf]

Appendix 1 – READ codes used for ADHD diagnosis

Read code v2	Description
Eu9y700	[X]Attention deficit disorder
ZS91.12	[X]Attention deficit disorder
Eu90011	[X]Attention deficit hyperactivity disorder
Eu90100	[X]Hyperkinetic conduct disorder
Eu90111	[X]Hyperkinetic disorder associated with conduct disorder
Eu90z00	[X]Hyperkinetic disorder, unspecified
Eu90.00	[X]Hyperkinetic disorders
Eu90z12	[X]Hyperkinetic syndrome NOS
Eu90y00	[X]Other hyperkinetic disorders
ZS91.11	ADD - Attention deficit disorder
ZS91.00	Attention deficit disorder
E2E0100	Attention deficit with hyperactivity
E2E0000	Attention deficit without hyperactivity
E2E0.00	Child attention deficit disorder
E2E0z00	Child attention deficit disorder
NOS E2E..00	Childhood hyperkinetic syndrome
E2E2.00	Hyperkinetic conduct disorder
E2Ez.00	Hyperkinetic syndrome
NOS 1BR..00	Reduced concentration
1BR0.11	Short attention span
Z7C5312	Short attention span
