# Supplementary material for: Determinants of inter-practice variation in ADHD diagnosis and stimulant prescribing: cross-sectional database study of a national surveillance network
Source: BMJ Evid Based Med. 2019 Feb 14;24(4):155–61. doi: 10.1136/bmjebm-2018-111133 (PMC6678046; doi:10.1136/bmjebm-2018-111133)
Supplement: Supplementary data [file bmjebm-2018-111133supp002.pdf]

Appendix 2 - EMIS and READ codes used for stimulant medications recommended at the time of our study (1st Jan - 31st December 2016)

Category	Read Code	Term198
Methylphenidate	dw1G.	MEDIKINET XL 30mg m/r capsules
Methylphenidate	dw1H.	MEDIKINET XL 40mg m/r capsules
Methylphenidate	dw1I.	CONCERTA XL 27mg m/r tablets
Methylphenidate	dw1J.	MEDIKINET 5mg tablets
Methylphenidate	dw1K.	MEDIKINET 10mg tablets
Methylphenidate	dw1L.	MEDIKINET 20mg tablets
Methylphenidate	dw1n.	METHYLPHENIDATE HYDROCHLORIDE 54mg m/r tablets
Methylphenidate	dw1F.	MEDIKINET XL 20mg m/r capsules
Methylphenidate	dw1E.	MEDIKINET XL 10mg m/r capsules
Methylphenidate	dw1D.	EQUASYM XL 30mg m/r capsules
Methylphenidate	dw1C.	EQUASYM XL 10mg m/r capsules
Methylphenidate	dw1B.	*TRANQUILYN 20mg tablets
Methylphenidate	dw1A.	*TRANQUILYN 10mg tablets
Methylphenidate	dw19.	*TRANQUILYN 5mg tablets
Methylphenidate	dw18.	CONCERTA XL 36mg m/r tablets
Methylphenidate	dw17.	CONCERTA XL 18mg m/r tablets
Methylphenidate	dw16.	EQUASYM XL 20mg m/r capsules
Methylphenidate	dw15.	*EQUASYM 10mg tablets
Methylphenidate	dw14.	*EQUASYM 20mg tablets
Methylphenidate	dw13.	*EQUASYM 5mg tablets
Methylphenidate	dw12.	RITALIN 10mg tablets
Methylphenidate	dw11.	METHYLPHENIDATE HYDROCHLORIDE 10mg tablets
Methylphenidate	dc1z.	DEXAMPHETAMINE SULPHATE 20mg m/r capsules
Methylphenidate	dc1y.	DEXAMPHETAMINE SULPHATE 12.5mg m/r capsules
Methylphenidate	dc1x.	DEXAMPHETAMINE SULPHATE 7.5mg m/r capsules
Methylphenidate	dc1w.	DEXAMFETAMINE SULFATE 5mg tablets

Methylphenidate	dc1v.	DEXAMFETAMINE SULFATE 1mg/mL oral solution
Methylphenidate	dc14.	*DUROPHET 20mg m/r capsules
Methylphenidate	dc13.	*DUROPHET 12.5mg m/r capsules
Methylphenidate	dc12.	*DUROPHET 7.5mg m/r capsules
Methylphenidate	dc11.	*DEXEDRINE 5mg tablets
Methylphenidate	dw1O.	MEDIKINET XL 60mg m/r capsules
Methylphenidate	dw1p.	METHYLPHENIDATE HYDROCHLORIDE 60mg m/r capsules
Methylphenidate	dw1Q.	MATORIDE XL 36mg m/r tablets
Methylphenidate	dw1R.	MATORIDE XL 54mg m/r tablets
Methylphenidate	dw1s.	METHYLPHENIDATE HYDROCHLORIDE 40mg m/r capsules
Methylphenidate	dw1T.	XENIDATE XL 36mg m/r tablets
Methylphenidate	dw1u.	METHYLPHENIDATE HYDROCHLORIDE 30mg m/r capsules
Methylphenidate	dw1v.	METHYLPHENIDATE HYDROCHLORIDE 36mg m/r tablets
Methylphenidate	dw1w.	METHYLPHENIDATE HYDROCHLORIDE 18mg m/r tablets
Methylphenidate	dw1x.	METHYLPHENIDATE HYDROCHLORIDE 20mg m/r capsules
Methylphenidate	dw1y.	METHYLPHENIDATE HYDROCHLORIDE 5mg tablets
Methylphenidate	dw1z.	METHYLPHENIDATE HYDROCHLORIDE 20mg tablets

**Category**	**EMIS Preparation Code**	**Preparation Description**	**Approved Name/ Description**
Dexamphetamine	ADM/38535NEMIS	Adderall Xr M/R capsules 5 mg	Adderall Xr
Dexamphetamine	ADM/38536NEMIS	Adderall Xr M/R capsules 30 mg	Adderall Xr
Dexamphetamine	ADM/38538NEMIS	Adderall Xr M/R capsules 25 mg	Adderall Xr
Dexamphetamine	ADM/38540NEMIS	Adderall Xr M/R capsules 20 mg	Adderall Xr
Dexamphetamine	ADM/38542NEMIS	Adderall Xr M/R capsules 15 mg	Adderall Xr
Dexamphetamine	ADM/38543NEMIS	Adderall Xr M/R capsules 10 mg	Adderall Xr
Dexamphetamine	AMM/38505NEMIS	Amfetamine And Dexamfetamine M/R capsules 5 mg + 5 mg	Amfetamine And Dexamfetamine
Dexamphetamine	AMM/38525NEMIS	Amfetamine And Dexamfetamine M/R capsules 7.5 mg + 7.5 mg	Amfetamine And Dexamfetamine

Dexamphetamine	AMM/38528NEMIS	Amfetamine And Dexamfetamine M/R capsules 10 mg + 10 mg	Amfetamine And Dexamfetamine
Dexamphetamine	AMM/38531NEMIS	Amfetamine And Dexamfetamine M/R capsules 12.5 mg + 12.5 mg	Amfetamine And Dexamfetamine
Dexamphetamine	AMM/38532NEMIS	Amfetamine And Dexamfetamine M/R capsules 15 mg + 15 mg	Amfetamine And Dexamfetamine
Dexamphetamine	AMM/38533NEMIS	Amfetamine And Dexamfetamine M/R capsules 2.5 mg + 2.5 mg	Amfetamine And Dexamfetamine
Dexamphetamine	AMTA105019NEMIS	Amfexa Tablets 5 mg	Amfexa
Dexamphetamine	AMTA117765NEMIS	Amfexa Tablets 20 mg	Amfexa
Dexamphetamine	AMTA117766NEMIS	Amfexa Tablets 10 mg	Amfexa
Dexamphetamine	DEM/16182NEMIS	Dexamphetamine Sulfate M/R capsules 15 mg	Dexamphetamine Sulfate
Dexamphetamine	DEM/17620NEMIS	Dexamfetamine M/R capsules 15 mg	Dexamfetamine
Dexamphetamine	DEOR21158NEMIS	Dexamfetamine Oral solution 5 mg/5 ml	Dexamfetamine
Dexamphetamine	DEOR45620NEMIS	Dexamfetamine Oral suspension 5 mg/5 ml	Dexamfetamine
Dexamphetamine	DEOR90855NEMIS	Dexamfetamine Oral Solution, Sugar Free 5 mg/5 ml	Dexamfetamine
Dexamphetamine	DESP16183NEMIS	Dexedrine Spansules 15 mg	Dexedrine
Dexamphetamine	DETA117763NEMIS	Dexamfetamine Tablets 10 mg	Dexamfetamine
Dexamphetamine	DETA117764NEMIS	Dexamfetamine Tablets 20 mg	Dexamfetamine
Dexamphetamine	DETA17621NEMIS	Dexamfetamine Tablets 5 mg	Dexamfetamine
Dexamphetamine	DETA3413	Dexedrine Tablets 5 mg	Dexedrine
Dexamphetamine	DETA4815	Dexamphetamine Sulfate Tablets 5 mg	Dexamphetamine Sulfate
Methylphenidate	COM/101185NEMIS	Concerta Xl M/R tablets 54 mg	Concerta Xl
Methylphenidate	COM/12672NEMIS	Concerta Xl M/R tablets 18 mg	Concerta Xl
Methylphenidate	COM/12674NEMIS	Concerta Xl M/R tablets 36 mg	Concerta Xl
Methylphenidate	COM/23819NEMIS	Concerta M/R tablets 27 mg	Concerta
Methylphenidate	COM/25051NEMIS	Concerta Xl M/R tablets 27 mg	Concerta Xl
Methylphenidate	DEM/120497NEMIS	Delmosart M/R tablets 18 mg	Delmosart
Methylphenidate	DEM/120498NEMIS	Delmosart M/R tablets 27 mg	Delmosart
Methylphenidate	DEM/120499NEMIS	Delmosart M/R tablets 36 mg	Delmosart
Methylphenidate	DEM/120500NEMIS	Delmosart M/R tablets 54 mg	Delmosart
Methylphenidate	EQM/11858NEMIS	Equasym Xl M/R capsules 20 mg	Equasym Xl

Methylphenidate	EQM/19955NEMIS	Equasym Xl M/R capsules 10 mg	Equasym Xl
Methylphenidate	EQM/19956NEMIS	Equasym Xl M/R capsules 30 mg	Equasym Xl
Methylphenidate	EQTA4119NEMIS	Equasym Tablets 5 mg	Equasym
Methylphenidate	EQTA4120NEMIS	Equasym Tablets 10 mg	Equasym
Methylphenidate	EQTA4121NEMIS	Equasym Tablets 20 mg	Equasym
Methylphenidate	MAM/95145NEMIS	Matoride Xl M/R tablets 36 mg	Matoride Xl
Methylphenidate	MAM/95147NEMIS	Matoride Xl M/R tablets 54 mg	Matoride Xl
Methylphenidate	MAM/96294NEMIS	Matoride Xl M/R tablets 18 mg	Matoride Xl
Methylphenidate	MEM/11856NEMIS	Methylphenidate Hydrochloride M/R capsules 20 mg	Methylphenidate Hydrochloride
Methylphenidate	MEM/12670NEMIS	Methylphenidate Hydrochloride M/R tablets 18 mg	Methylphenidate Hydrochloride
Methylphenidate	MEM/12673NEMIS	Methylphenidate Hydrochloride M/R tablets 36 mg	Methylphenidate Hydrochloride
Methylphenidate	MEM/15526NEMIS	Methylphenidate Hydrochloride M/R tablets 20 mg	Methylphenidate Hydrochloride
Methylphenidate	MEM/19953NEMIS	Methylphenidate Hydrochloride M/R capsules 10 mg	Methylphenidate Hydrochloride
Methylphenidate	MEM/19954NEMIS	Methylphenidate Hydrochloride M/R capsules 30 mg	Methylphenidate Hydrochloride
Methylphenidate	MEM/23817NEMIS	Methylphenidate Hydrochloride M/R tablets 27 mg	Methylphenidate Hydrochloride
Methylphenidate	MEM/24746NEMIS	Methylphenidate Hydrochloride M/R capsules 40 mg	Methylphenidate Hydrochloride
Methylphenidate	MEM/24747NEMIS	Medikinet Xl M/R capsules 10 mg	Medikinet Xl
Methylphenidate	MEM/24748NEMIS	Medikinet Xl M/R capsules 20 mg	Medikinet Xl
Methylphenidate	MEM/24749NEMIS	Medikinet Xl M/R capsules 30 mg	Medikinet Xl
Methylphenidate	MEM/24750NEMIS	Medikinet Xl M/R capsules 40 mg	Medikinet Xl
Methylphenidate	MEM/51599NEMIS	Methylphenidate Hydrochloride M/R capsules 5 mg	Methylphenidate Hydrochloride
Methylphenidate	MEM/51600NEMIS	Medikinet Xl M/R capsules 5 mg	Medikinet Xl
Methylphenidate	MEM/90836NEMIS	Methylphenidate Hydrochloride M/R capsules 50 mg	Methylphenidate Hydrochloride
Methylphenidate	MEM/90837NEMIS	Methylphenidate Hydrochloride M/R capsules 60 mg	Methylphenidate Hydrochloride
Methylphenidate	MEM/90838NEMIS	Medikinet Xl M/R capsules 50 mg	Medikinet Xl
Methylphenidate	MEM/90839NEMIS	Medikinet Xl M/R capsules 60 mg	Medikinet Xl
Methylphenidate	MEM/95146NEMIS	Methylphenidate Hydrochloride M/R tablets 54 mg	Methylphenidate Hydrochloride
Methylphenidate	MEOR30805NEMIS	Methylphenidate Hydrochloride Oral solution 5 mg/5 ml	Methylphenidate Hydrochloride
Methylphenidate	MEOR95955NEMIS	Methylphenidate Hydrochloride Oral suspension 5 mg/5 ml	Methylphenidate Hydrochloride

Methylphenidate	META23980EMIS	Methylphenidate Hydrochloride Tablets 10 mg	Methylphenidate Hydrochloride
Methylphenidate	META24742NEMIS	Medikinet Tablets 5 mg	Medikinet
Methylphenidate	META24743NEMIS	Medikinet Tablets 10 mg	Medikinet
Methylphenidate	META24744NEMIS	Medikinet Tablets 20 mg	Medikinet
Methylphenidate	META36026MGEMIS	Methylphenidate Hydrochloride Tablets 10 mg (A.A.H. Pharm)	Methylphenidate Hydrochloride
Methylphenidate	META36027MGEMIS	Methylphenidate Hydrochloride Tablets 10 mg (Unichem)	Methylphenidate Hydrochloride
Methylphenidate	META36028MGEMIS	Methylphenidate Hydrochloride Tablets 10 mg (Teva UK)	Methylphenidate Hydrochloride
Methylphenidate	META37714MGEMIS	Methylphenidate Hydrochloride Tablets 5 mg (A.A.H. Pharm)	Methylphenidate Hydrochloride
Methylphenidate	META37715MGEMIS	Methylphenidate Hydrochloride Tablets 20 mg (A.A.H. Pharm)	Methylphenidate Hydrochloride
Methylphenidate	META4114NEMIS	Methylphenidate Hydrochloride Tablets 5 mg	Methylphenidate Hydrochloride
Methylphenidate	META4116NEMIS	Methylphenidate Hydrochloride Tablets 20 mg	Methylphenidate Hydrochloride
Methylphenidate	RIM/12595NEMIS	Ritalin Sr M/R tablets 20 mg	Ritalin Sr
Methylphenidate	RITA23978EMIS	Ritalin Tablets 10 mg	Ritalin
Methylphenidate	TRTA13267NEMIS	Tranquilyn Tablets 5 mg	Tranquilyn
Methylphenidate	TRTA13268NEMIS	Tranquilyn Tablets 10 mg	Tranquilyn
Methylphenidate	TRTA13269NEMIS	Tranquilyn Tablets 20 mg	Tranquilyn
Methylphenidate	XAM/123300NEMIS	Xaggitin Xl M/R tablets 18 mg	Xaggitin Xl
Methylphenidate	XAM/123301NEMIS	Xaggitin Xl M/R tablets 27 mg	Xaggitin Xl
Methylphenidate	XAM/123302NEMIS	Xaggitin Xl M/R tablets 36 mg	Xaggitin Xl
Methylphenidate	XAM/123303NEMIS	Xaggitin Xl M/R tablets 54 mg	Xaggitin Xl
Methylphenidate	XEM/105347NEMIS	Xenidate Xl M/R tablets 54 mg	Xenidate Xl
Methylphenidate	XEM/115292NEMIS	Xenidate Xl M/R tablets 27 mg	Xenidate Xl
Methylphenidate	XEM/95188NEMIS	Xenidate Xl M/R tablets 18 mg	Xenidate Xl
Methylphenidate	XEM/95189NEMIS	Xenidate Xl M/R tablets 36 mg	Xenidate Xl
